# Associations of Chiari-1 malformation and syringomyelia with osseous cervical spinal canal diameter in the pediatric spine

**DOI:** 10.1007/s00381-026-07233-9

**Published:** 2026-03-27

**Authors:** Alexander T. Yahanda, Devin W. Kolmetzky, Vivek P. Gupta, Jennifer M. Strahle, Sean D. McEvoy, Gabe Haller, Thanda Meehan, Brian A. Kelly, Scott J. Luhmann, Joshua S. Shimony, James C. Torner, David D. Limbrick

**Affiliations:** 1 Department of Neurological Surgery, Washington University School of Medicine, St. Louis, MO USA; 2 Department of Orthopedic Surgery, Washington University School of Medicine, St. Louis, MO USA; 3 Department of Radiology, Washington University School of Medicine, St. Louis, MO USA; 4 Department of Neurological Surgery, School of Medicine, University of Iowa, Iowa City, IA USA; 5 Department of Neurological Surgery, Virginia Commonwealth University School of Medicine, Richmond, VA USA

**Keywords:** Cervical spine, Chiari-1 malformation, Pediatric spine, Syringomyelia, Spinal canal diameter, Spinal canal dilation, Spine development

## Abstract

**Purpose:**

To examine the associations between cerebellar tonsillar ectopia and syrinx size on bony spinal canal diameter in pediatric patients to explore the impact of CM with or without SM on the developing spine.

**Methods:**

Cohorts of patients with CM + SM, CM-only, and neither condition were compared. Anteroposterior (AP) syrinx diameter was measured at C2 (SX_2_) and C7 (SX_7_) for patients with syringomyelia. Tonsillar ectopia was measured for all patients in the CM + SM and CM-only cohorts. AP diameter of the bony spinal canal at C2 (SC_2_) and C7 (SC_7_) was measured for all patients. Patients were stratified by age and sex in secondary analyses.

**Results:**

357 patients had CM + SM and 217 patients had CM-only. Both cohorts had larger SC_2_ (p < 0.0001) and SC_7_ (p < 0.0001) compared to controls. CM + SM had larger SC_7_ (p < 0.0001) but not SC_2_ (p = 0.10) compared to CM-only. Increased SX_2_ was associated with increased SC_2_ (p < 0.0001); both SX_2_ and SX_7_ were significantly associated with increases in SC_7_ (p < 0.0001). Syringes ≥ 3 mm were associated with larger SC_2_ and SC_7_ compared to syringes < 3 mm (p ≤ 0.002). SC_2_ and SC_7_ for syringes < 3 mm were not significantly different from CM-only. Children with CM + SM in early childhood/adolescence experienced the greatest percentage spinal canal dilation. Females experienced greater spinal canal dilation than similarly-aged males.

**Conclusions:**

Patients with CM ± SM experience significant increases in bony cervical spinal canal diameter during development. Syringes ≥ 3 mm with CM are associated with further canal dilation of the spinal canal at syrinx level. The degree of spinal canal dilation appears to be age- and sex-dependent.

**Supplementary Information:**

The online version contains supplementary material available at 10.1007/s00381-026-07233-9.

## Introduction

Chiari-1 malformation (CM) is a common pediatric neurosurgical condition that is frequently associated with syringomyelia (SM) [1, 2]. Various studies report the incidence of SM in CM at 23–80% [3–5]. Approximately 20% of patients with CM also have spinal deformity (SD), though this may be as high as 70% in patients with both CM and SM [6]. While the associations between CM with or without SM and spinal abnormalities are well-described, the pathophysiology linking them remains poorly understood and the biological basis of this relationship has yet to be rigorously investigated.

Some clues to the physiologic links between these relationships may be found in comorbid conditions where CM, SM, and spinal anomalies are over-represented, such as Marfan’s syndrome and Neurofibromatosis type I, where remodeling of the vertebral bodies is often observed [7–9]. This remodeling may be related to changes in connective tissue, bone, dura or leptomeninges, or intradural physiology, which may evolve over time throughout development [9–11]. Thus, the interplay between the observed osseous remodeling in these conditions and concurrent physiologic changes at different points in development may provide insight into how the pediatric spine is affected over time by CM and/or SM.

To further explore the impact of CM with or without SM on the developing pediatric spine, we examined the associations between tonsillar ectopia and syrinx size on bony cervical spinal canal width for children ages 0–17 years old. To achieve this, we compared cervical spinal canal dimensions in subjects from 3 distinct cohorts (subjects with CM + SM, subjects with CM alone, and subjects with neither condition). We furthermore stratified these patients by age and sex to elucidate any potential interplay between timing of spinal development, degree of tonsillar ectopia, and syrinx size. These cohorts and comparisons make this study the first of its kind.

## Methods

Radiological data were obtained from three independent sources and grouped into three cohorts:

- *Cohort 1 (CM* + *SM)*: These data were obtained from the Park-Reeves Syringomyelia Research Consortium (PRSRC) registry (Table 1). The PRSRC is a multicenter research collaborative that acquired data both retrospectively (from July 2011 to October 2014) and prospectively (since October 2014), with a total of 42 contributing centers. All subjects had CM with cerebellar tonsillar ectopia ≥ 5 mm and syrinx diameter ≥ 3 mm. Subject demographic, clinical, and imaging data were de-identified with arbitrary site and subject numbers and recorded in a central database by clinical coordinators at each individual site under the oversight of a data monitor at the PRSRC’s host institution. Patients between the ages of 0–17 with T2-weighted preoperative cervical spine MRI studies were selected from the PRSRC registry. All images were collected after October 2001. For this study, preoperative baseline scans were used to conduct radiological measurements (Supplemental Fig. 1). All study-related procedures were approved by institutional review boards at the host institution (IRB 201011724) and each participating PRSRC center.
- *Cohort 2 (CM-only)*: Imaging studies were obtained from individuals managed at the authors’ institution with the diagnosis of CM *without* concurrent SM (Table 1). Individuals diagnosed with CM alone were identified via ICD-10 codes and were selected if they met the definition of CM (tonsillar ectopia ≥ 5 mm). As with Cohort 1, only one cervical MRI scan was analyzed per patient (Supplemental Fig. 1). Patients were excluded from this cohort if their imaging did not show true CM (i.e. tonsillar ectopia < 5 mm) or if they had SM. Subjects in this CM-only cohort were within the same age range (Table 1) and treatment interval as those in the PRSRC cohort. All images were collected after January 2007. Use of this cohort was approved by the institutional review board at the authors’ institution (IRB 201711041).
- *Cohort 3 (control; no CM or SM)*: This dataset of cervical spine morphometry was derived from measurements from cervical CT scans of 498 children with *neither* CM nor SM between the ages of 0–17 years that were analyzed and previously published by Johnson et al. [12]. The imaging studies used in this cohort were conducted between 2008 and 2013. In this study, only patients with normal CT scans were analyzed, which included patients with no evidence of congenital spine abnormality, prior spine surgery, or traumatic spine injury. The indications for CT imaging were not specified in this manuscript, nor was the percentage of patients who also underwent cervical MRI [12]. For our study, measurements were obtained directly from the Johnson et al. study.

**Table 1:** Demographic and clinical characteristics

Variable	No. patients (%)		
	**CM + SM (n = 357)**	**CM-only (n = 217)**	**P-value**
**Mean age (years ± SD)**	9.7 ± 5.2	8.9 ± 5.3	0.09
**Gender**			
Male	174 (48.7)	111 (51.1)	0.61
Female	183 (51.3)	106 (48.9)	
**Mean tonsillar ectopia (mm)**	12.8 ± 4.9	9.9 ± 4.6	** < 0.0001**
**Mean SC**_**2**_** (mm ± SD)**	17.4 ± 2.3	17.1 ± 2.0	0.10
**Mean SC**_**7**_** (mm ± SD)**	15.8 ± 2.0	14.8 ± 1.4	** < 0.0001**
**Mean SX**_**2**_** (mm ± SD)**	5.4 ± 3.1	–	–
**Mean SX**_**7**_** (mm ± SD)**	8.7 ± 3.6	–	–

CM = Chiari-1 malformation; CM + SM = Chiari-1 malformation with concurrent syringomyelia (SM); SC_2_ = spinal canal diameter of the C2 vertebrae; SC_7_ = spinal canal diameter of the C7 vertebrae; SD = standard deviation; ns = not statistically significant

Linear measurements of all preoperative cervical MRI studies were performed by a single-trained evaluator using the imaging software ITK-SNAP [13] with an image resolution of 0.01 mm. Maximum anteroposterior (AP) spinal canal diameter was measured at C2 (SC_2_) and C7 (SC_7_) using the methods described by Johnson et al. [12]. Maximum AP syrinx diameter was measured at C2 (SX_2_) and C7 (SX_7_) by drawing a line through the widest portion of the syrinx that was perpendicular to the vertical axis of the vertebra in question.

While all PRSRC subjects had syrinx measuring ≥ 3 mm, these were not all necessarily within the cervical spine. Thus, this study defined cervical syrinx to be present only if the AP diameter of that syrinx was ≥ 3 mm at that level. All patients in CM + SM and CM-only cohorts had tonsillar ectopia ≥ 5 mm (mean tonsillar ectopia in cohort 1: 12.8 ± 4.9 mm; cohort 2: 9.9 ± 4.6 mm).

To account for age-dependent changes in spinal growth patterns over the course of childhood development, radiological measures were categorized into four groups, which were separated by periods with different rates of spine and spinal canal growth [12]. The relationship between spine growth and skeletal maturity is complex, incompletely understood, and entertains reasonable difference in expert opinion [14–16]. This binning was based on biological maturation of the developing spine as has been previously described in multiple prior radiological studies, with female development progressing more rapidly than for males [12, 15, 17–21], as well as after reviewing with pediatric neurosurgery and orthopedic spine clinical experts. Bin #1 was comprised of patients from infancy to early childhood, during which there was significant vertical spinal growth. Bin #2 was comprised of patients from toddler age to puberty onset. Bin #3 consisted of patients who were undergoing puberty and concomitant rapid spine growth. Finally, Bin #4 included patients who had completed puberty and who had attained relative spinal maturity (through age 17 years) (Table 2). On average, females begin and finish puberty earlier than males [22]. Thus, binning periods for females were adjusted by one year. As this was a multi-institutional retro- and prospective observational study, we were not able to require radiographs to assess skeletal maturity. Our cohort also had an upper age limit of 17 years old, so further growth until 19–20 years of age was outside the scope of this paper.

**Table 2:** Binning strategy to account for natural age- and sex-dependent differences in bony spinal development. To adjust for natural age- and sex-dependent differences during childhood development, patients were separated into age-based bins. Age ranges were determined based on data from our control dataset,^20^ prior literature, and expert opinions

**Bin #1—Infancy to Early Childhood** Birth to around toddler age. There is significant vertical spinal growth in this period, with 50% occurring within the first year of life	Males: 0–4 years
	Females: 0–4 years
**Bin #2 – Later Childhood** Toddler age to onset of puberty. In this period, there is no significant increase in spinal canal diameter and there is minimal vertical spinal growth	Males: 5–10 years
	Females: 5–9 years
**Bin #3 – Adolescence** Beginning at the onset of puberty and ending with termination of puberty. This is the period encompassing the most rapid vertical growth of the spine during childhood development	Males: 11–15 years
	Females: 10–14 years
**Bin #4 – Adolescence to Adulthood** Comprised of children after cessation of puberty. Spinal maturity is being approached. While there may be some growth, it is not as rapid or of the same magnitude as during adolescence. The upper age limit in this study is 17 years of age	Males: 16–17 years
	Females: 15–17 years

Spinal dimensions from Cohort 1 were derived from CT images while all measurements from Cohorts 2 and 3 were derived from MRI studies. To evaluate for equivalence, 27 patients from the PRSRC cohort were identified who had both preoperative CT and MRI studies conducted within one month of each other. A single trained evaluator was responsible for making the measurements on MRI and CT for the same patients. They were not blinded but analyzed CT and MRI images in separate bundles such that they could be minimally influenced by any one set of measurements between imaging modalities.

Statistical analyses were performed using GraphPad Prism version 8.3.1 and SAS version 9.4. Student’s t-test was used to compare continuous variables with a 2-sided p-value < 0.05 for all tests being considered statistically significant.

## Results

In all, 357 patients were included in the CM + SM cohort (183 females, 51.3%). For the CM-only cohort, 217 patients were included (106 females, 48.8%). The mean age was 9.7 ± 5.2 years for the CM + SM cohort and 8.9 ± 5.3 years for the CM cohort. Demographic characteristics are listed in Table 1. There was not a statistically significant age difference between these two cohorts (p = 0.09). The two cohorts had significantly different mean tonsillar ectopia (CM + SM cohort: 12.8 ± 4.9 mm; CM-only cohort: 9.9 ± 4.6; p < 0.0001) and mean SC_7_ (CM + SM cohort: 15.8 ± 2.0; CM-only cohort: 14.8 ± 1.4; p < 0.0001).

**Fig. 1:**
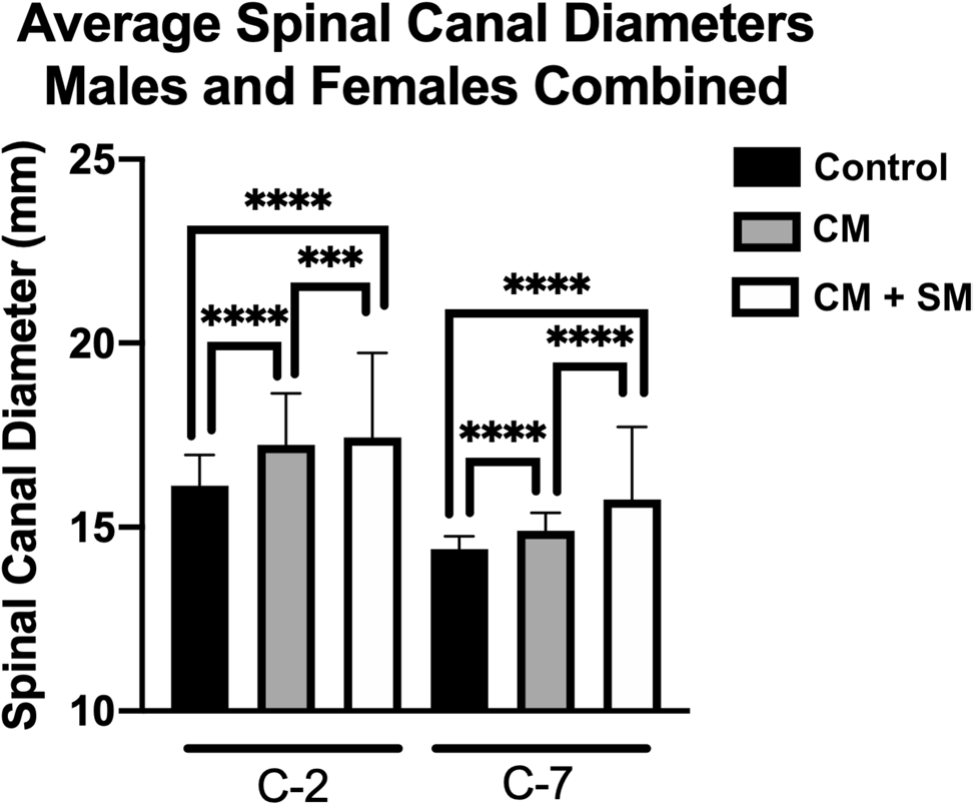
Average spinal canal diameters at C2 and C7 for each cohort

In considering all three cohorts, children with CM + SM had significantly larger SC_2_ and SC_7_ compared to children from the control cohort (Table 3), demonstrating an average increase of 8.12% for SC_2_ (p < 0.0001) and 9.44% for SC_7_ (p < 0.0001) (Fig. 1). Children with CM alone also had significantly larger SC_2_ and SC_7_ compared to children from the control cohort, showing an average increase of 6.88% for SC_2_ (p < 0.0001) and 3.47% for SC_7_ (p < 0.0001) (Fig. 1). While children with CM + SM did not have significantly larger average SC_2_ compared to children with CM alone, those with CM + SM had significantly larger average SC_7_ (p < 0.0001).

**Table 3:** CM and SM are associated with significantly increased size of SC_2_ (**a**) and SC_7_ (**b**) compared to control group. Compared to CM-only, patients with CM + SM had greater SC7 (b) but not at SC_2_ (a)

**A**		Control	CM-only	CM + SM (all syringes)
**SC**_**2**_	n	498	217	359
	Average Age (Years)	9.63	8.32	9.22
	SD (Age)	5.98	8.32	9.22
	Average SC_2_ (mm)	16.13	17.24	17.44
	SD (SC_2_)	1.62	1.94	2.28
	Control vs. CM	p < 0.0001		****
	Control vs. CM + SM	p < 0.0001		****
	CM vs. CM + SM	p = 0.282		ns
**B**		Control	CM-only	CM + SM (all syringes)
**SC**_**7**_	n	498	217	359
	Average Age (Years)	9.63	8.32	9.22
	SD (Age)	5.98	5.04	5.22
	Average SC_7_ (mm)	14.40	14.90	15.76
	SD (SC_7_)	1.23	1.31	1.96
	Control vs. CM	p < 0.0001		****
	Control vs. CM + SM	p < 0.0001		****
	CM vs. CM + SM	p < 0.0001		****

CM = Chiari-1 malformation; CM + SM = Chiari-1 malformation with concurrent syringomyelia (SM); SC_2_ = spinal canal diameter of the C2 vertebrae; SC_7_ = spinal canal diameter of the C7 vertebrae; SD = standard deviation; ns = not statistically significant

On univariate linear regression analyses, increases in both SX_2_ (p < 0.0001) and SX_7_ (p = 0.01) were significantly associated with increased SC_2_. When including both SX_2_ and SX_7_ together in multivariate analysis, only SX_2_ (p = 0.001) significantly impacted SC_2_ (SX_7_ p = 0.19). On univariate regression, increases in both SX_2_ (p < 0.0001) and SX_7_ (p < 0.0001) significantly impacted SC_7_. On subsequent multivariate analysis, both these variables remained significantly associated with increased SC_7_ (SX_2_ p = 0.04; SX_7_ p < 0.0001).

**Fig. 2:**
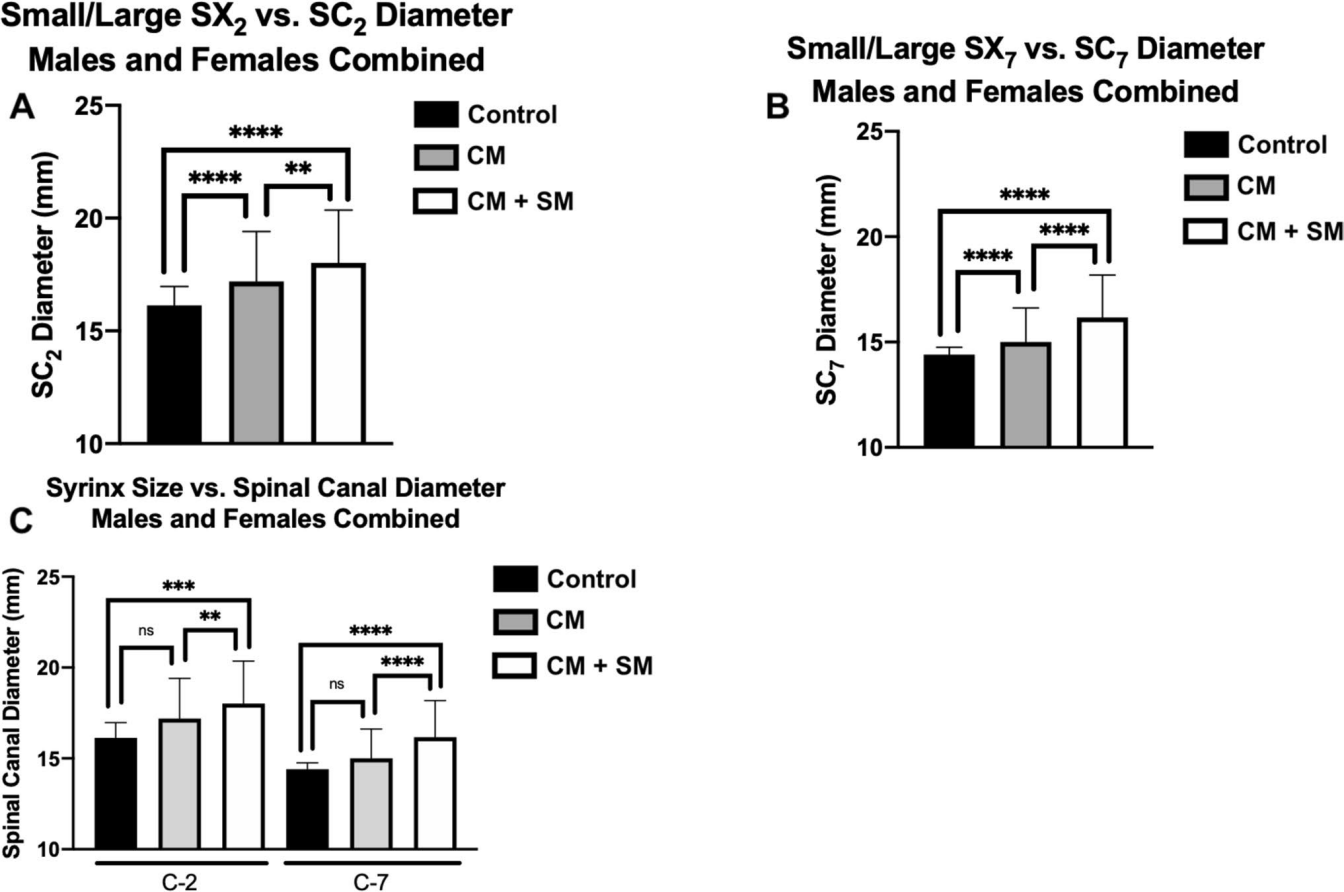
Effect of syrinx size on spinal canal diameter at C2 (**a**), C7 (**b**), and compared to patients with CM-only (**c**)

In the CM + SM cohort a syrinx was deemed present at a particular level only if its diameter was ≥ 3 mm. We compared patients with ≥ 3 mm syringes to patients with syringes at that same level that were < 3 mm to evaluate the effect of syrinx size on spinal canal diameter. Syringes ≥ 3 mm in AP diameter were associated with significantly larger cervical spinal canal diameters (at the level of their syrinx) than syringes < 3 mm at that same level (Table 4). Children with SX_2_ ≥ 3 mm showed an average increase of 11.72% in SC_2_ (p < 0.0001) compared to the control cohort, while children with SX_2_ < 3 mm showed an average increase of 6.63% in SC_2_ at that level (p < 0.0001) (Fig. 2). At C7, this difference was more pronounced: children with SX_7_ ≥ 3 mm showed a 12.29% mean increase in SC_7_ at that level (p < 0.0001) compared to the control cohort while children with SX_7_ < 3 mm showed a 4.17% mean increase in SC_7_ at that level (p < 0.0001) (Fig. 2). In fact, the amount of SC_2_ and SC_7_ dilation at any given level in children with syrinx < 3 mm was indistinct from children with CM-only (p = 0.836 and 0.535, respectively) (Fig. 2). There was no significant relationship between the degree of tonsillar ectopia and SX_2_ or SX_7_.

**Table 4:** At both C2 (**a**) and C7 (**b**), CM + SM patients with SM ≥ 3 mm have a greater spinal canal diameter compared to control cohort, CM-only cohort, and CM + SM cohort with SM < 3 mm

**A**		Control	CM-only	CM + SM (SX_2_ < 3 mm)	CM + SM (SX_2_ ≥ 3 mm)
SC_2_	n	498	217	253	106
	Average Age (Years)	9.63	8.32	8.43	11.11
	SD (Age)	5.98	8.32	5.24	4.65
	Average SC_2_ (mm)	16.13	17.24	17.20	18.02
	SD (SC_2_)	1.62	1.94	2.21	2.34
	Control vs. CM			p < 0.0001	****
	Control vs. CM + SM (SX_2_ < 3 mm)			p < 0.0001	****
	Control vs. CM + SM (SX_2_ ≥ 3 mm)			p < 0.0001	****
	CM vs. CM + SM (SX_2_ < 3 mm)			p = 0.282	ns
	CM vs. CM + SM (SX_2_ ≥ 3 mm)			p = 0.002	**
	CM + SM (SX_2_ < 3 mm) vs CM + SM (SX_2_ ≥ 3 mm)			p = 0.002	**
**B**		Control	CM-only	CM + SM (SX_7_ < 3 mm)	CM + SM (SX_7_ ≥ 3 mm)
SC_7_	n	498	217	124	235
	Average Age (Years)	9.63	8.32	9.10	9.29
	SD (Age)	5.98	5.04	5.62	5.00
	Average SC_7_ (mm)	14.40	14.90	15.00	16.17
	SD (SC_7_)	1.23	1.31	1.62	2.01
	Control vs. CM			p < 0.0001	****
	Control vs. CM + SM (SX_7_ < 3 mm)			p < 0.0001	****
	Control vs. CM + SM (SX_7_ ≥ 3 mm)			p < 0.0001	****
	CM vs. CM + SM (SX_7_ < 3 mm)			p = 0.535	ns
	CM vs. CM + SM (SX_7_ ≥ 3 mm)			p < 0.0001	****
	CM + SM (SX_7_ < 3 mm) vs CM + SM (SX_7_ ≥ 3 mm)			p < 0.0001	****

CM = Chiari-1 malformation; CM + SM = Chiari-1 malformation with concurrent syringomyelia (SM); SC_2_ = spinal canal diameter of the C2 vertebrae; SC_7_ = spinal canal diameter of the C7 vertebrae; SD = standard deviation; ns = not statistically significant

**Fig. 3:**
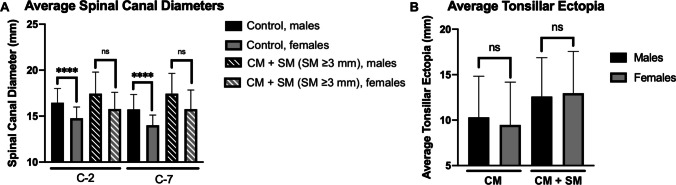
Sex-dependent differences in the spinal canal dilation

The severity of spinal canal dilation seen in patients with CM with or without SM exhibited sex-dependence, with females demonstrating more severe dilation. Within the control cohort, males had significantly larger SC_2_ and SC_7_ than females (p < 0.0001 for both); however, in children with syrinx > 3 mm, males and females showed no statistically significant differences in SC_2_ (p = 0.967) and SC_7_ (p = 0.962) (Fig. 3A). There were no sex-dependent differences in tonsillar ectopia in children with CM with or without SM (Fig. 3B).

**Fig. 4:**
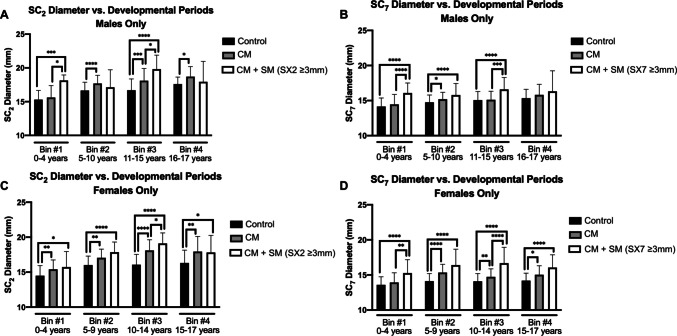
Age-dependent differences in spinal canal dilation

Children with imaging demonstrating CM + SM in early childhood and adolescence were associated with the greatest percentage spinal canal dilation. In early childhood, males with CM + SM presented with 18.55% larger SC_2_ (Fig. 4A) and 13.64% larger SC_7_ (Fig. 4B). However, in late childhood, the difference in SC_2_ was not significant (2.82%) while the difference in SC_7_ was only 7.05%. During adolescence, males presented with 18.68% larger SC_2_ and 10.09% larger SC_7_, but in post-pubertal males, SC_2_ or SC_7_ were not significantly different.

Similar to males, adolescent females with CM + SM (Figs. 4C and 4D) displayed the largest percent increase in SC_2_ and SC_7_ (18.86% and 18.30%, respectively). Unlike males, however, females with CM + SM in late-childhood and post-puberty had larger SC_2_ and SC_7_ compared to normative dimensions, but these measurements were not significantly larger compared to CM alone. Females with CM + SM in early childhood showed 8.56% larger SC_2_ (not significant compared to CM-only) and 12.36% larger SC_7_.

On subgroup analysis of the patients who had both CT and MRI scans, no significant differences and strong correlations were identified between the two imaging modalities (p = 0.61, r = 0.74 for SC_2_; p = 0.77, r = 0.75 for SC_7_), and variances for comparisons were randomly distributed.

## Discussion

Our study is the most thorough illustration of an association between syrinx size and location and bony vertebral canal diameter seen in the cervical spine of children diagnosed with CM + SM. While changes in cervical spinal canal tapering ratios have been observed in patients with CM and SM [23–26], this is the first study to isolate and quantify Chiari- and syrinx-dependent spinal canal dilation occurring throughout pediatric spinal development and to correlate the degree of dilation with syrinx size and location. We have demonstrated that cervical spine syringes are associated with bony spinal canal enlargement in the region of the syrinx. This syrinx-associated dilation diminishes at increasing distances from the syrinx and is modified by factors such as female sex and age.

It is possible that the more significant relationships between SM and spinal canal diameters at C7 versus at C2 are a consequence of cerebellar tonsils competing for space with the syrinx in the rostral cervical spine, thereby limiting the maximum syrinx size. Because CM was shown to be associated with spinal canal dilation (more substantially at SC_2_ than at SC_7_) independent of SM, the statistical significance of the relationship between SX_2_ and SC_2_ may be dampened by baseline Chiari-related SC_2_ dilation. At the level of C7, the relationship between SX_7_ and SC_7_ diameters has stronger statistical significance. However, further studies should be performed to verify these findings.

It should be noted that the metrics we used to describe the relationship between SM and spinal canal diameter are two-dimensional metrics based on cross-sectional slices. While a relationship between these two metrics has been demonstrated in this study, the next logical investigative step would be to examine three-dimensional (3D) volume of the syrinx, spinal cord, and spinal canal. This could be crudely performed with a mathematical volumetric calculation or, with the advent of increasingly powerful computer-assisted segmentation tools, be performed using automated volumetric analysis using a region of interest on imaging software. We hypothesize that the two-dimensional relationships illustrated in this paper would be magnified when evaluated in 3D.

The association between SM and spinal deformity is well-documented [27–29] but the mechanism behind this association is ill-defined. In children < 17 years old diagnosed with scoliosis, those with concurrent SM exhibit left-sided curvature far more commonly than right-sided curvature [30]. The relationship between SM and scoliotic curve progression is inconsistent, with some studies reporting that patients with wider/longer synriges or holocord syringes demonstrate higher rates of scoliosis [27, 31] and others finding less strong associations between scoliosis and syrinx/position or tonsillar descent [30, 32]. The specific 3D morphological effects of these changes in spinal cord and canal diameter are outside the scope of this paper and will be the subject of future study. Our study demonstrates that cervical spine syringes are associated with regional spinal canal dilation, highlighting the potential presence of a mechanism by which SM or related physiological processes induce bony changes. It is possible that these mechanisms may share similar underlying biology with adolescent idiopathic scoliosis. The mechanism of Chiari-induced SM, as with the relationship between SM and spinal deformity, is highly nuanced, with no consensus mechanism to describe syrinx formation [33, 34]. There are several theories that have been put forth involving CSF pressure differentials, pulsatility, or hydrodynamic forces, though no single hypothesis has been definitively proven.

At birth, the spine is mostly cartilaginous. Through precisely-timed changes in growth factors and gonadal steroids during development [14], it gradually reaches ~ 60% ossification at 10 years [35] of age and becomes fully ossified in early adulthood. Johnson et al. observed that while cervical spinal canal diameter did not significantly increase after 4 years of age, spinal dimensions continued to shift until ~ 17 years old in males and ~ 14 years old in females [12]. This suggests that the variability in the change in canal diameter we observed may occur as a function of differing bony compliance at different ages. These findings are also consistent with remodeling of bone that is seen in conditions involving dural ectasia – for instance, neurofibromatosis type 1 and Marfan’s syndrome – highlighting the roles that CSF pulsatility or thecal sac dynamics may have on different connective tissue biology. Similarly, while the link between connective tissue disorders such as the Ehlers-Danlos Syndromes and bony changes is still under investigation, changes in bone mineral density in these patients and the increased incidence of vertebral abnormalities suggest that the interplay with connective tissue during development could also contribute to the changes we observed [36, 37].

Our observation of sex-dependent differences in spinal canal dilation seems intuitive given the sex-dependent differences in developmental growth patterns [12, 30]. In addition, females are more likely to have an associated syrinx when diagnosed with CM and are more likely to require hospitalization for Chiari- and SM-related symptoms [3]. Our observation that females’ predisposition to larger syringes despite having smaller average spinal canals at baseline supports the notion that the degree of spinal canal dilation perhaps associated with the severity of Chiari- and SM-related symptoms, but investigating this potential phenomenon is outside the scope of the current study. Regarding the age binning strategy employed in this paper, multiple different rating systems for stage of skeletal maturity have been developed, including the Sanders score, Risser score, and evaluation of the triradiate cartilage [15, 16]. Despite this, there remains discrepancy between remaining skeletal growth and how it predicts the development of spinal pathology (e.g. adolescent idiopathic scoliosis) [14, 16]. Given these discrepancies, we took a combined approach to separate ages by aggregated growth velocity after reviewing a variety of expert opinions and in consultation with our pediatric neurosurgery and orthopedic spine surgeon co-authors.

The management of CM and SM is a complex issue with various proposed treatment strategies and propensity for surgical complications [38, 39]. Most pediatric neurosurgeons, however, tend to offer surgical management CM-1 with SM, even if asymptomatic [40]. Longitudinal studies of patients with SM who do not receive surgery show more inconsistent outcomes, ranging from partial-to-complete syrinx resolution to rapid syrinx progression with devastating neurological consequences [3, 41, 42].

Since the management of CM + SM is nuanced and complicated, several metrics have been developed to inform preoperative decision-making (e.g. clivo-axial angle, C-C2SVA, pB-C2 distance, or other measurements of skull base and upper cervical spine anatomy) [43]. Fewer metrics involve the cervical spine itself, particularly the lower cervical spine, and how CM with or without SM may impact these areas. Some spinal deformities (e.g. scoliosis) have been shown to worsen if left uncorrected during periods of substantial growth, especially during early childhood and adolescence [31, 44]. Furthermore, previous work has demonstrated that syringomyelia and scoliosis have the highest chance of response to surgery at an earlier age and, in the case of scoliosis, with less severe curvature [31]. Our study showed that CM + SM has the potential to exert age- and sex-dependent effects on the diameter of the developing cervical spine. If a syrinx harbors the capacity for spinal canal dilation, our observations provide another metric for further characterization of individuals with CM + SM that may be used to track disease progression and guide clinical counseling. Additionally, this would merit investigation into a more nuanced picture of what potential spinal canal changes may be imparted by CM alone versus CM + SM. Our findings, then, provide an important foundation on which further studies may be based. While at present, these findings should not prompt any specific changes to the management of CM + SM, they set the stage for additional investigation into the physiological basis of bony spine development and related clinical conditions that require surgical treatment.

It is an interesting point to consider that, if the CM and/or SM are sufficiently addressed by surgery, the spinal canal may experience differential growth and development now that potential contributing factors to canal dilation have been mitigated. Nevertheless, we considered postoperative changes to canal diameter to be outside of the scope of this specific paper. We primarily sought to demonstrate relationships between CM, SM, and canal diameter to establish that such associations were even present. This is certainly something that is of interest to our group, and subsequent steps could examine the effect that surgical intervention may have on future canal diameter.

### Limitations

Patients included in this study were ≤ 17 years old; thus, these findings are not necessarily transferrable to adults with CM ± SM or males whose spine may continue to grow beyond 17 years. Syrinx-dependent spinal canal dilation could presumably also occur in the adult spine, as well as in the thoracic or lumbar spine, but these analyses were beyond the scope of this paper. These ideas certainly warrant further investigations.

Furthermore, the number of available patients in our study potentially skewed statistical significance. There were very few children < 4 years old and > 16 years old with CM + SM (specifically those with SX_2_ ≥ 3 mm). The small number of children < 4 years old in the PRSRC database may be attributed to the inherent presentation of CM + SM in young children or relatively lower rates of MRI usage in this age range due to need for sedation. Lack of children > 16 years may be due to the small number of older children included in this study as well as age-dependent clinical presentations [45]. Moreover, further measurement techniques for canal diameter would add nuance to our understanding of any relationships between CM, SM, and canal diameter of the developing spine. However, for the measurement of CM and SM, not all patients had axial measurements. Variations in scanning parameters and protocols across PRSRC sites also prohibited a more formal volumetric analysis of either bony spinal canal or syrinx sizes.

Notably, any morphometric study based on analyses of imaging is subject to measurement error, and measurements of the pediatric cervical spine may be affected by variable degrees of ossification [12]. Differences in windowing between CT and MRI can lead to measurement differences that may alter results. Spinal dimensions from the control cohort were derived from CT images while all measurements from patients in the PRSRC and SLCH cohorts were derived from MRI studies, which presents a limitation in our ability to compare measurements between modalities. It is possible that mild dural ectasia could have influenced canal diameter, though we did not observe other associated findings such as scalloping of vertebral bodies or thinning of ribs to support dural ectasia. Therefore, we feel confident that our findings were representative of true canal widening.

Though we found no significant differences in spinal canal measurements between CT versus MRI, this subgroup analysis was conducted on a relatively small percentage of the overall number of patients who were included in our study, as there were a limited number of patients who had both CT and MRI scans. In an ideal scenario, a control cohort consisting of patients who had received an MRI would have been created. However, as mentioned in the Johnson et al. paper [12], MRI scans in children are more difficult to obtain than in adults due to the need for sedation. Thus, crafting cohorts of pediatric patients of many different ages who all had normal MRI scans would be challenging and may not reach adequate numbers for analysis. Johnson et al. had created an excellent baseline repository of normal cervical spine morphometrics, so we compared our two cohorts to their data, even though their measurements were derived from CT. Certainly, moving forward, the development of an MRI-only repository for normative cervical spine measurements will be of keen interest for these authors. Of note, all comparisons between the CM and CM + SM cohorts in this study were performed using solely MRI scans.

## Conclusions

In pediatric patients with CM + SM, presence of CM and SM were associated with differing degrees of bony cervical spinal canal dilation. The association between syringes and canal dilation dissipated with increasing distance from the ectopic tonsils and/or syrinx and compounded when tonsils and syrinx were in proximity. Females with CM + SM displayed greater susceptibility to spinal canal dilation than males. Age also appeared be a factor for the degree of spinal canal dilation, with the greatest degrees of dilation observed in children of ages in which the spine is naturally undergoing the most substantial developmental changes.

## Supplementary Information

Below is the link to the electronic supplementary material.

Supplementary file1 MRI images demonstrating measurements of tonsillar ectopia and canal width at C2 (SC2) and C7 (SC7) (left) as well as measurements of syrinx AP diameter at C2 (SX2) and C7 (SX7) (right) (PDF 3472 KB)

Supplementary file2 (DOCX 22 KB)

## Data Availability

The data underlying this study are derived from the multicenter Park-Reeves Syringomyelia Research Consortium, institutional data from Saint Louis Children's Hospital, and institutional data from the University of Michigan. De-identified data may be available from the corresponding author on reasonable request and subject to various institutional approvals.

## Declarations

None of the authors have anything to disclose.
