# Supplementary figures and images for: Associations of Chiari-1 malformation and syringomyelia with osseous cervical spinal canal diameter in the pediatric spine
Source: Childs Nerv Syst. 2026 Mar 27;42(1):137. doi: 10.1007/s00381-026-07233-9 (PMC13021709; doi:10.1007/s00381-026-07233-9)

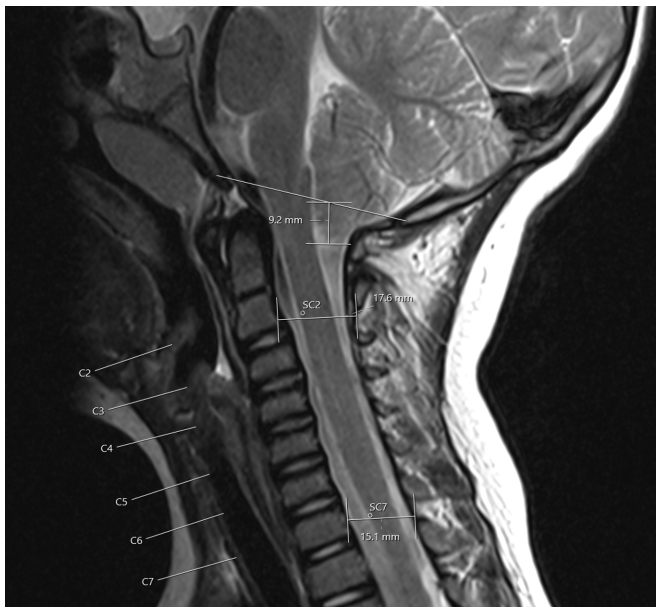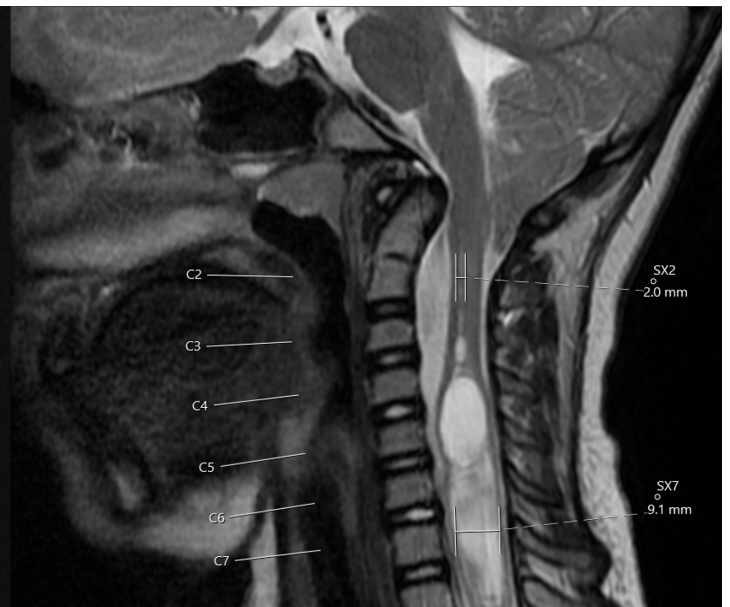

Supplement: Supplementary file 1 — Supplementary file1 MRI images demonstrating measurements of tonsillar ectopia and canal width at C2 (SC2) and C7 (SC7) (left) as well as measurements of syrinx AP diameter at C2 (SX2) and C7 (SX7) (right) (PDF 3472 KB) [file 381_2026_7233_MOESM1_ESM.pdf]
