# Supplementary material for: Associations of Chiari-1 malformation and syringomyelia with osseous cervical spinal canal diameter in the pediatric spine
Source: Childs Nerv Syst. 2026 Mar 27;42(1):137. doi: 10.1007/s00381-026-07233-9 (PMC13021709; doi:10.1007/s00381-026-07233-9)
Supplement: Supplementary file 2 — Supplementary file2 (DOCX 22 KB) [file 381_2026_7233_MOESM2_ESM.docx]

|  |  | MRI | CT |
| --- | --- | --- | --- |
| C2 | n | 27 | 27 |
|  | Average SC_2_ (mm) | 17.29 | 17.05 |
|  | SD | 1.91 | 1.57 |
|  | MRI vs. CT | p = 0.6161 | ns |
| C7 | n | 25 | 25 |
|  | Average SC_7_ (mm) | 15.18 | 15.29 |
|  | SD | 1.35 | 1.33 |
|  | MRI vs. CT | p = 0.7729 | ns |

**Supplement Table 1.** There were no statistically significant differences in spinal canal diameter measurements when performed on CT or T2-weighted MRI.

SC_2_ = spinal canal diameter of the C2 vertebrae; SC_7_ = spinal canal diameter of the C7 vertebrae; SD = standard deviation; ns = not statistically significant
